# Nanoscale synchrotron X-ray speciation of iron and calcium compounds in amyloid plaque cores from Alzheimer's disease subjects†
†Electronic supplementary information (ESI) available. See DOI: 10.1039/c7nr06794a. Raw images and spectral data for this paper will be accessible *via* the Keele Research Repository at http://dx.doi.org/10.21252/KEELE-0000027.


**DOI:** 10.1039/c7nr06794a

**Published:** 2018-04-24

**Authors:** James Everett, Joanna F. Collingwood, Vindy Tjendana-Tjhin, Jake Brooks, Frederik Lermyte, Germán Plascencia-Villa, Ian Hands-Portman, Jon Dobson, George Perry, Neil D. Telling

**Affiliations:** a Institute for Science and Technology in Medicine , Thornburrow Drive , Keele University , Staffordshire , ST4 7QB , UK; b Warwick Engineering in Biomedicine , School of Engineering , Library Road , University of Warwick , Coventry , CV4 7AL , UK . Email: J.F.Collingwood@warwick.ac.uk; c Department of Materials Science and Engineering , University of Florida , Gainesville , FL 32611 , USA; d Department of Physics and Astronomy. The University of Texas at San Antonio (UTSA) , San Antonio , TX 78249 , USA; e School of Life Sciences , Gibbet Hill Campus , University of Warwick , Coventry , CV4 7AL , UK; f J. Crayton Pruitt Family Department of Biomedical Engineering , Institute for Cell and Tissue Science & Engineering , University of Florida , Gainesville , FL 32611 , USA; g Department of Biology and UTSA Neurosciences Institute. The University of Texas at San Antonio (UTSA) , San Antonio , TX 78249 , USA

## Abstract

Altered metabolism of biometals in the brain is a key feature of Alzheimer's disease, and biometal interactions with amyloid-β are linked to amyloid plaque formation. Iron-rich aggregates, including evidence for the mixed-valence iron oxide magnetite, are associated with amyloid plaques. To test the hypothesis that increased chemical reduction of iron, as observed *in vitro* in the presence of aggregating amyloid-β, may occur at sites of amyloid plaque formation in the human brain, the nanoscale distribution and physicochemical states of biometals, particularly iron, were characterised in isolated amyloid plaque cores from human Alzheimer's disease cases using synchrotron X-ray spectromicroscopy. *In situ* X-ray magnetic circular dichroism revealed the presence of magnetite: a finding supported by ptychographic observation of an iron oxide crystal with the morphology of biogenic magnetite. The exceptional sensitivity and specificity of X-ray spectromicroscopy, combining chemical and magnetic probes, allowed enhanced differentiation of the iron oxides phases present. This facilitated the discovery and speciation of ferrous-rich phases and lower oxidation state phases resembling zero-valent iron as well as magnetite. Sequestered calcium was discovered in two distinct mineral forms suggesting a dynamic process of amyloid plaque calcification *in vivo*. The range of iron oxidation states present and the direct observation of biogenic magnetite provide unparalleled support for the hypothesis that chemical reduction of iron arises in conjunction with the formation of amyloid plaques. These new findings raise challenging questions about the relative impacts of amyloid-β aggregation, plaque formation, and disrupted metal homeostasis on the oxidative burden observed in Alzheimer's disease.

## Abstract 2

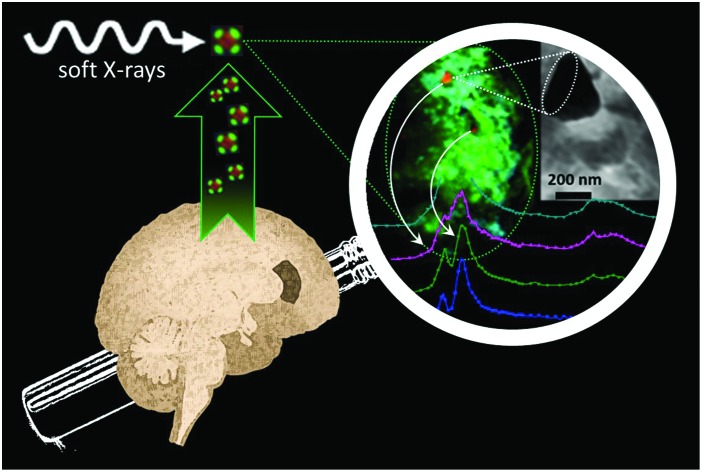
Synchrotron soft X-ray nano-imaging and spectromicroscopy reveals iron and calcium biomineralization in Alzheimer's disease amyloid plaques.

## Introduction

Disrupted metal ion homeostasis has been linked to the development and progression of Alzheimer's disease (AD). Calcium is the most abundant metal element in the human brain, and disturbed calcium signalling pathways and elevated intracellular calcium levels have been reported in conjunction with AD pathogenesis.^1^–^3^ Transition metals are present at trace levels in comparison to calcium, but they still play many essential roles in normal brain metabolism. Of the transition metals associated with AD pathology, iron is the most abundant in the healthy brain and is critical for normal brain function.

In the AD brain, iron concentration is significantly elevated in several tissue regions including the putamen.^4^ Locally elevated concentrations of atypical iron oxide aggregates and evidence of neurotoxic redox-active iron phases correlate with pathological hallmarks of AD throughout the brain. Sophisticated regulatory processes are required to maintain homeostasis, trafficking and storage for the many biometals essential to neuronal function.^5^ For example, iron is essential in energy production, nerve impulse transduction and neurotransmitter synthesis;^5^,^6^ these roles are enabled through controlled iron valence state changes *in vivo*, with both ferric (Fe^3+^) and ferrous (Fe^2+^) iron normally present in the brain. Non-heme iron is stored as a comparatively *redox-inactive* ferrihydrite-like mineral typically of the form (5Fe_2_O_3_·9H_2_O), a ferric oxyhydroxide phase within the 12 nm protein cage ferritin.^5^,^7^ Iron binding is protective against iron partaking in redox reactions (*e.g.* Fenton chemistry) which may overwhelm antioxidant defences with the excess generation of reactive oxygen species (ROS).^8^ The most chemically available ‘labile’ and *redox-active* form is ferrous iron which may comprise ∼5% of total intracellular iron.^9^ Redox-active iron levels are understood to be tightly regulated by oxidation–reduction (redox) processes such as the ferroxidase function of ferritin.^5^,^10^ Likewise, calcium (Ca) is vital for brain function and it plays fundamental roles in the development and plasticity of the nervous system. A large gradient exists between extracellular (10^–3^ M) and intracellular Ca^2+^ (10^–7^ M) pools, maintained by active pumping of Ca^2+^ through channels in the cell membrane.^11^ Maintaining these gradients enables cells to use transient increases in intracellular calcium concentrations as an initiation event for a variety of cellular responses, including: neurotransmitter release, metabolic regulation, cell growth, synaptic efficiency and long-term potentiation. Therefore the maintenance of both calcium and iron homeostasis in brain is fundamental to its normal function, with metal dysregulation being shown to have catastrophic effects.^11^–^14^

Iron dysregulation has been implicated in the development of AD, an age-related neurodegenerative condition which is the most common cause of dementia amongst the elderly.^15^ The underlying causes of the disease are not fully understood, and no effective treatments or cure exist. Evidence of significant cell damage, in conjunction with markers of oxidative stress, has resulted in oxidative damage being investigated as a major effector of neurodegeneration.^16^–^18^ Increased levels of material incorporating ferrous iron, potentially capable of catalysing redox chemistry have been reported post-mortem in AD subjects compared to age-matched disease-free controls.^19^–^22^ It is therefore possible that increased redox-active iron loading in AD provides a source of oxidative stress. As iron accumulation and oxidative stress have been shown as early events in AD,^23^ the presence of inappropriate levels of redox-active iron could be a key event in triggering Aβ aggregation and free radical damage in AD.

Although the origin of the ferrous iron associated with AD is unclear, evidence implicates amyloid-β (Aβ) in this phenomenon.^17^,^24^–^28^ Aβ is the major constituent of amyloid plaque cores (APC),^29^ a hallmark lesion of AD that is understood to convey neurotoxicity directly through its ability to produce reactive species including ROS,^30^,^31^ and indirectly by inducing the formation of neurofibrillary tangles (NFTs, comprised of hyper-phosphorylated tau protein).^32^,^33^ There are numerous reports of iron-containing Aβ plaques, including some reports that plaques incorporate ferrous-rich phases (such as the magnetic iron oxide, magnetite [Fe_3_O_4_]), as evidenced by histochemical staining,^21^ microscopic particle-induced X-ray emission analysis (microPIXE),^34^ MRI,^35^ HR-TEM and 3D electron tomography.^36^ Furthermore, Aβ plaques have been shown to be associated with ferritin in AD,^37^ and ferritin isolated from AD post-mortem brain was reported to contain increased levels of ferrous iron compared to controls.^38^ These observations indicate that Aβ is associated with the formation of phases incorporating ferrous iron by altering the way iron is handled. Indeed, the ability of Aβ to directly alter iron chemistry has been demonstrated previously. *In vitro* studies showed that Aβ can induce the redox-cycling of iron precipitates,^26^ while our previous X-ray absorption studies showed that Aβ chemically reduces a variety of ferric iron phases (including ferrihydrite) into pure ferrous forms.^24^,^25^ The conversion of redox-inactive iron into redox-active phases has the potential to cause significant oxidative damage to neuronal populations; therefore, targeting amyloid/iron interaction in AD may prove an effective means to lower overall oxidative stress and delay disease progression.

Another factor indicated in the development of AD is disrupted calcium signalling.^1^,^39^,^40^ Perturbed intracellular calcium homeostasis induced signal-transduction cascades associated with AD, mutations in genes associated with familial AD showed a direct effect on calcium homeostasis, and calcium was implicated as a co-factor in the formation of Aβ plaques and NFTs,^1^ suggesting that Aβ may be directly involved in disrupted calcium handling. Transgenic mice displaying amyloid deposition displayed impaired calcium homeostasis,^39^ whilst *in vitro* studies showed that addition of Aβ to cell cultures induced an influx of calcium across the cell membrane.^41^ Levels of Ca^2+^ are higher in aged neurons, which may reflect compromised management of calcium gradients across the cell membranes.^42^ Calcium has been observed in amyloid deposits isolated from the thalamus of transgenic APP mice and the hippocampus of human AD cases,^43^ whilst increased calcium levels have also been observed in human tissues displaying amyloid pathology.^44^,^45^ Some forms of calcium dysregulation may be a compensatory process in AD to modulate neuronal excitability and slow pathology.^42^

In our most recent investigation we provided *ex vivo* evidence demonstrating both ferrous iron and magnetite to be directly correlated with the presence of APC in cortical tissue from a transgenic mouse model of AD.^46^ However, the ability of Aβ to induce chemical reduction and biomineralisation of iron within human AD tissues is unproven, and the species of calcium and ferrous-rich iron phases associated with APC are not precisely described. Following the suggestion that the source of Aβ, the amyloid precursor protein APP, has a fundamental role in normal brain iron trafficking,^47^ it is even more critical that Aβ aggregates are considered in the context of brain metal ion (dys)metabolism. Understanding the chemical speciation of metals associated with AD pathology is crucial in the development of therapies intended to diagnose, monitor and treat the disorder. Identifying iron species and mineral phases disproportionately associated with AD could support clinical diagnosis using non-invasive iron-sensitive techniques such as MRI. Better understanding of iron species associated with AD may also facilitate the development of novel therapies intended to alleviate iron-associated toxicity.^48^ There are challenges to overcome if iron is to be selectively chelated for therapeutic purposes without compromising iron trafficking required for healthy brain function.

The aim of the present study was to investigate the distribution and physicochemical properties of iron-rich deposits in human APC, using a non-destructive spectromicroscopy technique that has not previously been applied to studies of human APC, Scanning Transmission X-ray Microscopy (STXM). This method has unique potential for precise determination of the nanoscale distribution and speciation of trace levels of organic and inorganic material in APC.

## Experimental methods

This study used spectromicroscopy with high spatial and energy resolution to determine the physicochemical characteristics of iron deposits in human APC. The technique of choice was STXM, a synchrotron-based X-ray technique which offers outstanding sensitivity to probe and subsequently map chemical speciation at spatial resolutions routinely approaching 20 nm. Further to this, STXM X-ray Circular Magnetic Dichroism (XMCD) was performed to elucidate the magnetic state of iron inclusions identified within APC.

### Isolation of amyloid plaque core (APC) material

Human brain tissue was obtained with the informed written consent of relatives, from autopsy AD patients. The protocols used to obtain, isolate and identify the APCs were approved by the Bioethics Committee (Department of Pathology, Case Western Reserve University), and this study was performed under UK ethical approval 07/MRE08/12 and USA IRB 03-00-26.

Brains from two AD cases (Braak stage VI) were removed at autopsy (5 h post-mortem) divided in half, cut into one cm slices and stored at –70 °C. Frozen tissue sections were thawed and grey matter from the frontal and temporal lobes was isolated by removing blood vessels, meninges and white matter. Grey matter was then homogenized by heating to 95 °C in the presence of 2% SDS and 50 mM Tris-buffer, before being filtered (110 μm pore size) to remove large tissue debris. The resulting material was pelleted through centrifugation at 800 rpm. AD tissues were further homogenized through the addition of 0.1% SDS, 150 mM NaCl and 0.02% NaN_3_ before being filtered (35 μm pore size) and pelleted at 1000 rpm for 15–30 min. APC were isolated from the 35 μm filtrate through ultracentrifugation at 20 000 rpm in a sucrose gradient (1.8–1.2 M sucrose, in a 0.1% SDS, 150 mM NaCl and 0.02% NaN_3_ solution). The resulting interfaces were collected and recovered for a final time with 0.1% SDS, 150 mM NaCl and 0.02% NaN_3_, before being concentrated through centrifugation at 1200 rpm.

### Embedding and Sectioning of APC

Approximately 40 μL of pelleted APC was transferred into a centrifugal concentrator (Corning® Spin-X® UF; 40 kDa cut-off) and was spun at 6690 rpm for 10 minutes. APC were dehydrated through an ethanol series (100 μL; 40%–100% dry), with waste ethanol being removed through centrifugation (6690 rpm for 10 minutes). Other chemical fixatives were not introduced, to limit metal ion leeching or transformation of mineral compounds. Once dehydrated, APC were embedded in a STXM compatible aliphatic resin comprised of a 1 : 1 molar mixture of trimethylolpropane triglycidyl ether : 4,4′-methylenebis(2-methylcyclohexylamine). This resin contains no carbonyl or aromatic groups, making it an ideal embedding substrate when examining protein-based structures, due the lack of strong spectral features at the carbon K-absorption edge that overlap with protein peaks. Resin was polymerized over 24 h at 60 °C.

Semi-thin sections containing APC were cut to a thickness of either 500 nm or 200 nm using a Reichert-Jung Ultra-cut microtome. A 500 nm thickness was cut to maximise the probability of observing APC, whereas a 200 nm thickness was cut to allow carbon spectroscopy using STXM. Non-metallic blades were used throughout the sectioning process to prevent metal contamination in the sample material.

### Congo red staining

Sections 500 nm in thickness from both AD cases were stained for amyloid structures using a 1% Congo red solution. Sections were stained for approximately 30 minutes, and excess stain was removed with deionized H_2_O. Stained sections were examined for birefringence under cross-polarized light using an Olympus IX51 microscope (60× objective lens).

### Scanning transmission X-ray microscopy

X-ray spectromicroscopy experiments were performed at the Advanced Light Source (Lawrence Berkeley National Laboratory, Berkeley CA, USA), on beamline 11.0.2 using the STXM end-station, and Diamond Light Source, (Oxfordshire, UK) on beamline I08, with a focused X-ray spot size of *ca.* 25 nm (25 nm zone plate) in both instances. Energy-specific images were created by raster scanning the sample across the focussed beam and recording transmitted X-ray intensity. Exposure times were kept to a minimum (≤5 ms per point) to prevent X-ray induced photo-reduction of APC constituents, based on our previous experiments using various iron standard samples.^46^

To map distributions of chemical species associated with APC, paired images were taken at the energy corresponding to the spectral feature of interest and an off-peak energy a few eV below the feature. The off-peak image was then subtracted to create a difference map, revealing the chemical speciation image over the region of interest for the selected spectral feature.

X-ray absorption spectra were obtained from a series of images (a ‘stack’) taken at energies spanning a desired absorption edge. The signal intensity recorded in the stack was converted to optical density using background regions that did not contain any sample material. For carbon K-edge absorption spectra, the background signal from the resin was subtracted as described in Telling *et al*.^46^ This method of spectromicroscopy allows X-ray absorption spectra to be generated from each pixel of an image, enabling spectral analysis of highly localised regions of interest. For stack measurements, the dark count (background noise attributable to the beamline) was subtracted prior to the generation of the X-ray absorption spectra.

XMCD experiments were performed at Diamond Light source on beamline I08 using circularly polarised light. XMCD measurements were obtained by attaching NdFeB permanent ring magnets (allowing X-ray transmission) to the back face of the sample probe, with a sample section being mounted to the front face. The magnetic field at the sample position was ∼150 mT, which is below the saturation field for magnetite, but sufficient to allow a degree of magnetic polarization. XMCD spectra were obtained by performing stacks over the iron L_3_-absorption edge (700–716 eV) using both right and left circularly polarised X-rays, with an exposure time of 5 ms per pixel. XMCD spectra were created by subtracting X-ray absorption spectra obtained under right circularly polarised (RCP) light from equivalent spectra obtained under left circularly polarised light (LCP). In addition to the APC material, an embedded synthetic magnetite nanopowder reference sample was created using the same embedding series as for the APC. APC and magnetite samples were prepared in separate laboratories so no cross contamination of samples could occur.

STXM data were processed using the aXis 2000 software package (http://unicorn.mcmaster.ca/aXis2000.html). For iron L_3_-edge X-ray absorption spectra collected for XMCD analysis, a 3-point smoothing filter was applied to the raw data. ImageJ software was used to adjust the brightness and contrast of X-ray microscopy images. Pseudo-coloured composite images were created by converting grey scale X-ray microscopy images to false colour before recombining the images as overlays. Twelve plaque cores were examined using X-ray spectromicroscopy.

### Analysis of X-ray absorption spectra

To obtain an estimate of the relative proportion of different iron phases within iron inclusions, reference X-ray absorption spectra from standards of Fe^3+^, Fe^2+^, Fe_3_O_4_ and Fe^0^ were used to fit the measured iron L_2,3_-edge X-ray absorption spectra from each area, using a non-linear least-squares fitting procedure. A precise quantitative determination of the phase proportions would require the accurate scaling of the X-ray absorption spectra from these reference samples. However, the required scaling factors are not easy to determine when comparing iron phases with oxidation states that vary from pure metal to Fe^3+^, due to the widely varying spectral shape and post-edge absorption intensities. Approximate scaling was instead determined by normalising the X-ray absorption intensity for each reference material (after background subtraction) to the integrated intensity over the L_2,3_ absorption edges (Fig. S1†), following a method similar to that discussed in Regan *et al*.^49^ However, we note here that the reference spectra we have used have only a limited post L_2_ range, and so the scaling is less accurate than would be obtained using more extended spectra. Despite this limitation, the estimated iron phase proportions derived in this way give a reliable indication of the relative differences between the individual iron-rich regions measured in the different inclusions.

We note that no evidence of chemical reduction was observed in ferric iron standards prepared using the same embedding series as for the APC, as described in the ESI† of our previously published work from the APP/PS1 mouse model of Alzheimer's disease (see Telling *et al*.^46^).

### Ptychography

Ptychography images of resin-embedded APC (500 nm thickness) were collected at the ALS beamline 11.0.2 using the STXM end-station. Paired images of the APC were taken at a peak iron L_3_-edge absorption energy (710 eV) and an off peak energy (705 eV) allowing an iron ptychography contrast map to be created.

## Results

### Identification of amyloid plaque cores (APC)

**Fig. 1:**
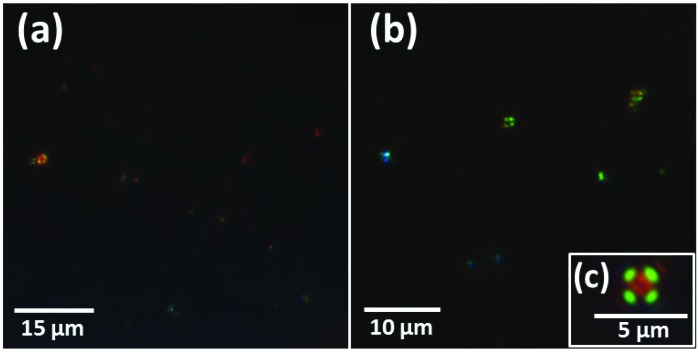
Congo red stained sections containing APC exhibiting birefringence under cross-polarized light. Sections (a) and (b) were obtained from case X, whilst section (c) was taken from case Y.

To confirm the presence and structural arrangement of APC in resin-embedded sections, Congo red staining was performed on 500 nm thick sections obtained from both AD cases. These were examined for birefringence using cross-polarizing optical microscopy. In both cases abundant areas with “apple-green” birefringence were observed (Fig. 1), confirming the presence and, importantly, the preservation of the fibrillar arrangement of APC. Radially symmetric birefringence, characteristic of spherulite amyloid structures^50^ (see Fig. 1c) was also observed throughout all sections examined, further verifying the presence of amyloid material.

### Iron and calcium loading of APC

The spatial distribution and chemical composition of APC in unstained 200 nm and 500 nm thick sections were determined using STXM. All resin-embedded sections in this study utilised a specialist aliphatic embedding resin that does not contain strong spectral features at the C, N or O absorption K-edges, differentiating it from commonly-used electron microscopy epoxy resins.^51^ We have elsewhere demonstrated how the contribution to the X-ray absorption from the resin can be subtracted from images obtained at the carbon absorption K-edge, in order to map a specific protein or peptide distribution in embedded sections of mouse brain tissue.^46^ The analytical procedure from the tissue analysis was applied to the isolated APC in the present study. Additionally, in the thicker 500 nm sections (which impede X-ray transmission at the principal carbon K-edge absorption energy of 288.5 eV for proteins), the oxygen K-edge was used to identify peptide (here amyloid) content from APC, as well as to identify other oxygenated compounds. Validation for the use of the oxygen K-edge in these thicker sections is demonstrated in ESI Fig. S2,† where near-equivalent peptide maps were obtained using protein X-ray absorption features at the carbon K-edge (285.0 eV) and the oxygen K-edge (532.1 eV).

The oxygen K-edge was ultimately chosen over the carbon K-edge for peptide mapping in 500 nm thick sections, as this absorption edge was far less susceptible to X-ray saturation effects.

**Fig. 2:**
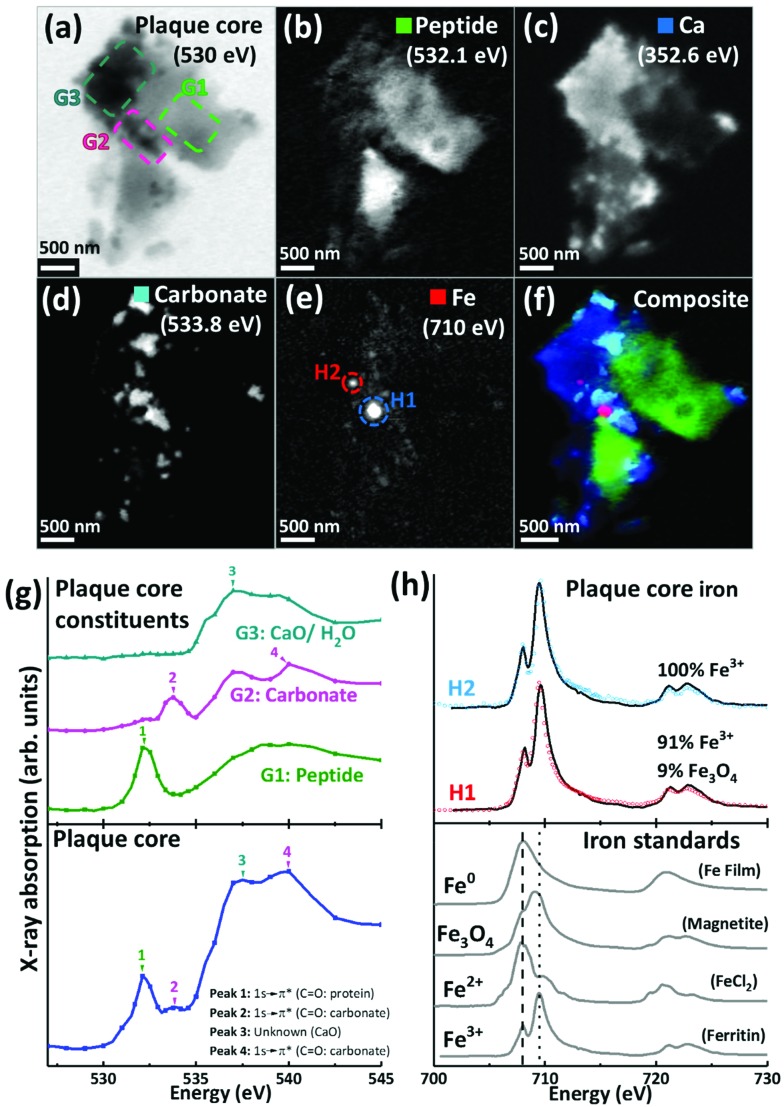
X-ray spectromicroscopy images and speciation dependent contrast maps, oxygen K-edge X-ray absorption spectra, and iron L_2,3_-edge X-ray absorption spectra, from an amyloid plaque core of case Y. (a) Off resonance 530 eV image. (b) Oxygen K-edge peptide map. (c) Calcium L-edge map. (d) Oxygen K-edge carbonate map. (e) Iron L-edge map. (f) Composite image displaying peptide (green), calcium (blue), carbonate (sky blue) and iron (red) content of the plaque core. (g) Oxygen K-edge X-ray absorption spectra from the whole sectioned plaque core (bottom), and localised plaque core regions (top) as identified in the 530 eV microscopy image (a). (h) Iron L_2,3_-edge X-ray absorption spectra from reference Fe^3+^ [ferritin], Fe^2+^ [FeCl_2_] and magnetite [Fe_3_O_4_] and iron zero [Fe^0^] standards (bottom), and two amyloid plaque core iron deposits (top) as labelled in the iron contrast map (e). The dashed line at 708 eV and dotted line at 709.5 eV in the iron reference spectra show the peak absorption energies for Fe^2+^ and Fe^3+^ cations respectively.

For the 500 nm thick sections, an X-ray beam energy of 530 eV (which is near the oxygen K-edge, but not associated with any spectral features) was used to observe the entire APC. Images obtained at this energy showed the APC to be dense granular structures ranging from 5 to 25 μm in size (see ESI Fig. S3†). To determine precisely the energies of the spectral features observed near the oxygen K-edge, an image stack was collected over the 525 eV–545 eV energy range from a typical APC (*ca.* 3.5 μm in diameter) as shown in Fig. 2a. The resulting X-ray absorption spectrum (Fig. 2g, lowest trace) included four main features: (1) a sharp peak at 532.1 eV, corresponding to 1 s to π* transitions from protein carbonyl groups;^52^ (2) a shallow peak at 533.8 eV, characteristic of 1 s to π* transitions from the carbonyl groups of carbonates;^53^ (3) a peak at 537.6 eV, attributed to calcium oxide (CaO) bonds;^53^ and finally (4) a 1 s to σ* carbonyl group carbonate peak at 540 eV (ref. 53) (although there was insufficient information in this energy region to define the exact position of this particular peak).

By examining localised regions of interest within this APC (highlighted as coloured boxes in Fig. 2a; top left), its heterogeneous nature was revealed with different constituents dominating the oxygen spectra in these selected regions (Fig. 2g; top). For example, the area labelled G1 (Fig. 2a) showed a single spectral feature at the oxygen K-edge at 532.1 eV (Fig. 2g) which was attributed to peptides. The map obtained from this spectral feature (Fig. 2b) showed strong intensity in the region of G1, which did not appear in the other maps (Fig. 2c–e). Of particular note, area G2 showed a strong feature at 533.8 eV attributed to carbonates (Fig. 2g, peak 2), and the corresponding map obtained using this feature (Fig. 2d) revealed the presence of small dense clusters. These carbonate-containing clusters were associated with the spectral feature from calcium oxide bonds (CaO) at 537.6 eV, and they can also be observed in the Ca map (Fig. 2c), suggesting them to be calcium carbonate. Interestingly, further areas of the APC demonstrating CaO absorption features (Fig. 2g, area G3) did not also show carbonate X-ray absorption features, indicating diversity in the calcium species present, beyond calcium carbonate. It should also be noted that water could contribute to the absorption feature at 537.6 eV (ref. 54) (peak 3 in Fig. 2g), which would be consistent with the presence of a hydrous calcium phase such as hydroxyapatite.

The APC displayed an inhomogeneous peptide distribution as evidenced by dense peptide spots and striations (Fig. 2b). A high level of calcium loading was observed throughout the APC (Fig. 2c), with some smaller (100–500 nm) localised regions of carbonate and iron deposition also present (Fig. 2d). Importantly, while carbonate was only found in APC regions containing calcium, there were calcium-rich regions that did not contain carbonate, indicating that not all calcium in APC was present as a carbonate phase. Alternative forms might be calcium divalent ions and calcium phosphate phases such as the aforementioned hydroxyapatite.

Iron L_3_-edge mapping showed iron to be present as well-defined dense clusters, and also distributed throughout the whole APC at a lower concentration (Fig. 2e). The two dense iron-containing regions (labelled H1 and H2 in Fig. 2e) were examined at a series of energies across the iron L_2,3_ absorption edge (700 eV–740 eV) to provide X-ray absorption spectra allowing the oxidation state of the iron to be elucidated. In order to provide an estimate of the relative proportions of the iron phases contributing to each iron L_2,3_-edge X-ray absorption spectrum measured in the APC, a non-linear least-squares fitting procedure was employed. Equivalent iron L_2,3_-edge X-ray absorption spectra from iron reference standards exhibiting different known oxidation states are presented in Fig. 2h (bottom), and scaled iron references used for fitting are shown in Fig. S1,† to facilitate accurate characterization of iron-rich inclusions in the APC. From the fits shown in Fig. 2h, both areas H1 and H2 contained predominantly ferric (Fe^3+^) iron, with H2 being a pure ferric phase (Fig. 2h, top).

**Fig. 3:**
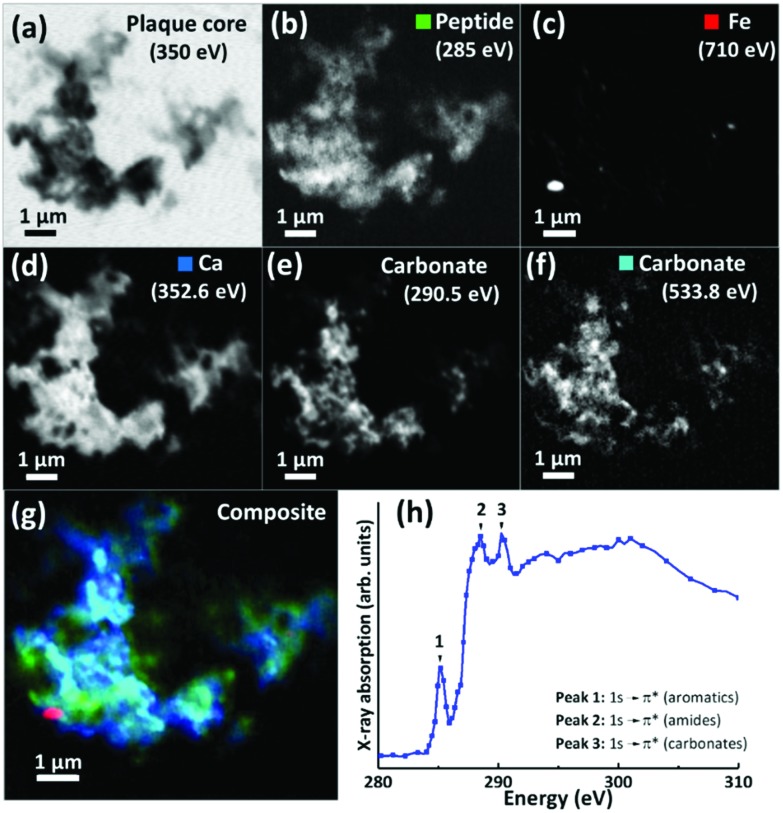
(a–g) X-ray microscopy images and speciation dependent contrast maps of an amyloid plaque core from case X (scale bar = 1 μm). (a) Off resonance calcium L-edge (350 eV) image. (b) Carbon K-edge protein map. (c) Iron L-edge map. (d) Calcium L-edge map. (e) Carbon K-edge carbonate map. (f) Oxygen K-edge carbonate map (g) Composite image showing: protein (green), calcium (blue), oxygen K-edge carbonate (sky blue), and iron (red) content of the plaque core. (h) Amyloid plaque core carbon K-edge X-ray absorption spectrum. Energy positions for 1 s to π* transitions for aromatics, amides and carbonates are labelled 1, 2 and 3 respectively.

For 200 nm thick sections, with an example shown in Fig. 3, an energy near the calcium L-edges (350 eV) was used to identify APC features (a) and the carbon K-edge was used to determine the spatial distribution of the peptide (Fig. 3b). In this example the calcium L-edge (as opposed to the carbon K-edge) was preferred for initial identification of APC, as a weaker contrast was obtained at the carbon edge due to carbon background signal in the embedding resin. As before, serial images were collected over the entire carbon K-edge (280–320 eV) to establish the exact position of carbon-based absorption features in the energy spectrum. The carbon K-edge X-ray absorption spectrum from the APC (shown in Fig. 3h) was comprised of a 3-peak structure, with two sharp peaks at (1) 285 eV and (2) 288.5 eV, corresponding to the 1 s to π* transitions of peptide aromatic and amide groups respectively, and a further peak (3) at 290.5 eV corresponding to the 1 s to π* transition of carbonate.^55^ The peak (1) at 285 eV was chosen for chemical mapping of peptide content, owing to its sharp energy resolution, and its lower absorption intensity compared to the high intensity peak (2) at 288.5 eV; with this higher energy peak being particularly vulnerable to X-ray saturation effects due to the strong absorption of 288.5 eV photons by both the resin and peptide.

Using these characteristic spectral features, species-specific spatial distribution maps within APC were generated as shown in Fig. 3, including peptide (285 eV, Fig. 3b), iron (710 eV, Fig. 3c), calcium (352.6 eV, Fig. 3d) and carbonate (290.5 and 533.8 eV, Fig. 3e and f respectively). As in Fig. 2, the APC is seen to be comprised of peptides varying in thickness/density resulting in intense protein spots, although these features were less apparent than in the 500 nm sections where more APC material was present (see ESI Fig. S4† for comparisons of peptide maps from 200 nm and 500 nm thick sections).

**Fig. 4:**
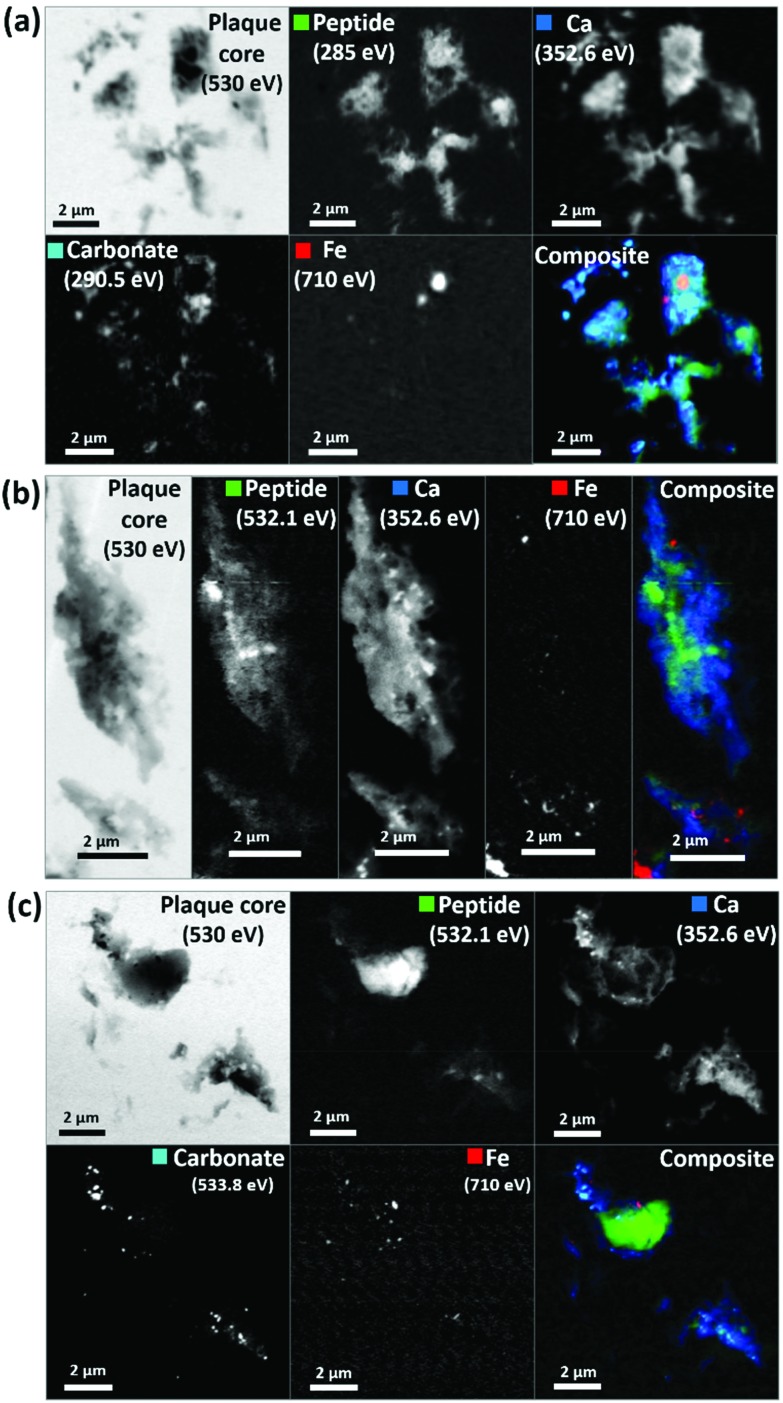
STXM images and speciation dependent contrast maps of amyloid plaque cores from case X (a), and case Y (b and c). The maps show the off-resonance (530 eV) image, oxygen K-edge peptide map (532 eV), calcium L-edge map (352 eV), oxygen K-edge carbonate map (533 eV), iron L-edge map (710 eV), and composite image displaying peptide (green), calcium (blue), carbonate (sky blue) and iron (red) content of the plaque core.

As in our first observations, the APC was heavily loaded with calcium (Fig. 3d). To confirm that large areas of carbonate were present throughout the plaque, corresponding maps were taken at carbonate spectral features using both the carbon K-edge (290.5 eV, Fig. 3e) and oxygen K-edge (533.8 eV, Fig. 3f) showing consistent contrast features, thus confirming the presence of carbonate. The carbonate distribution was confined to areas also containing calcium whilst the calcium content extends beyond this, indicating multiple calcium forms to be present. Further deposits of iron (*ca.* 200–500 nm diameter) were identified throughout this core (Fig. 3c), the oxidation states of which are considered in the following section. Additional examples of APC examined from both AD patient cases (labelled as case X and case Y) are shown in Fig. 4, confirming that *the same pattern* of concurrent calcium and iron loading was observed within the peptide rich amyloid cores. This pattern was repeated for *all* APC examined (see also ESI Fig. S5†). Furthermore, the identification of overlapping yet distinct calcium and calcium carbonate content was consistently observed throughout APC from both AD cases.

### Nanoscale iron distribution, oxidation and magnetic state

**Fig. 5:**
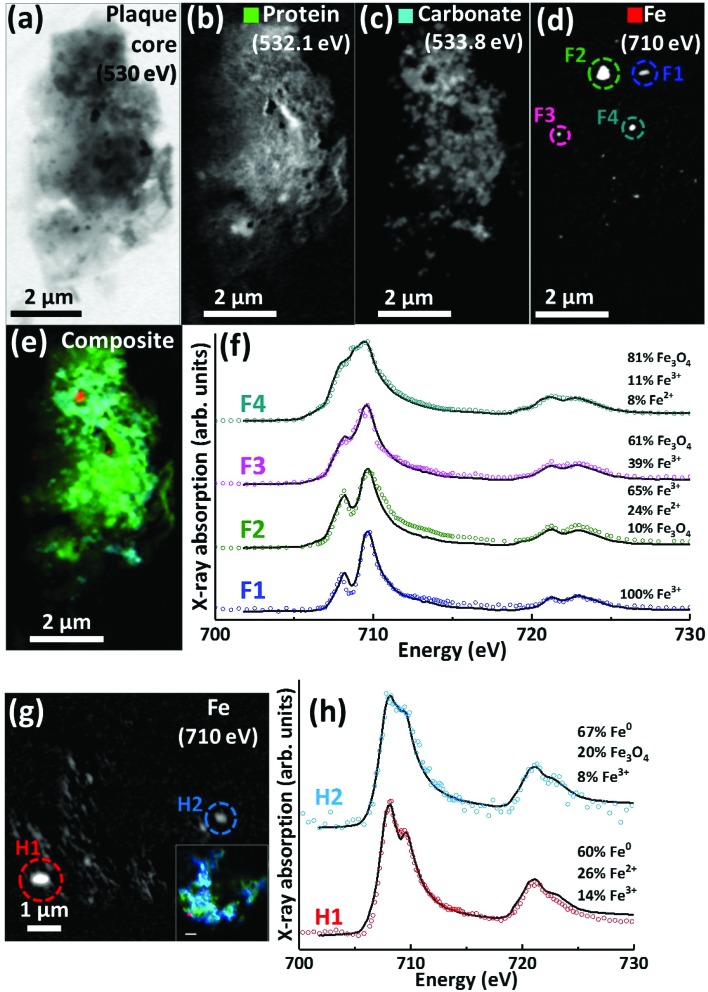
STXM images, speciation dependent contrast maps and iron L_2,3_-edge absorption spectra from an amyloid plaque from case Y (a–f) and case X (g–h). (a) Off resonance 530 eV image. (b) Oxygen K-edge protein map. (c) Oxygen K-edge carbonate map. (d) Iron L-edge map. (e) Composite image showing: protein (green), carbonate (sky blue) and iron (red). Scale bars = 2 μm. (f) Iron L_2,3_-edge absorption spectra from the iron deposits labelled in the iron map (d). (g) Iron L-edge map and composite (inset) of the amyloid plaque core shown in Fig. 3 (scale bar = 1 μm). (h) Iron L_2,3_-edge absorption spectra form the iron deposits labelled in the iron map (g). The solid lines for the spectra correspond to best fit curves created by superposition of suitably scaled iron reference X-ray absorption spectra see Fig. S1.†

Scanning transmission X-ray microscopy (STXM) images and speciation-dependent contrast maps of APC from case Y are displayed in Fig. 5(a–e). As with the previously described plaques, the APC was comprised of dense areas of peptides (Fig. 5b), exhibiting regions of carbonate (Fig. 5c) and iron deposition (Fig. 5d). This structure was too dense for calcium mapping at 352.6 eV. Examination of the iron deposits (highlighted in Fig. 5d) across the iron L_2,3_ absorption edge revealed iron to be present in ferric and ferrous-rich states (Fig. 5f). Area F1 appeared as a pure Fe^3+^ material with a principal iron L_3_ peak at 709.5 eV and a separate low energy feature at 708 eV, both arising from Fe^3+^ cations (as also seen in the Fe^3+^ spectra in Fig. 2h). Area F2 displayed features consistent with an increase of the ferrous iron containing phases Fe^2+^ and Fe_3_O_4_, as evidenced by the enhancement of the feature at 708 eV in relation to the principal Fe^3+^ peak at 709.5 eV. As the principal Fe^2+^ peak resides at 708 eV (see for example the ferrous chloride standard in Fig. 2h), this suggests a slight increase in Fe^2+^ content. The presence of Fe_3_O_4_ (*ca.* 10%) in area F2 was determined through the relative intensities of the iron L_2_-edge Fe^2+^ and Fe^3+^ absorption features (found at 721 eV and 723 eV respectively), which were approximately equal in intensity. Fitting showed that an increase in 708 eV peak intensity arising solely through the presence of Fe^2+^ resulted in a poor L_2_ edge fit, with the peak feature at 721 eV dominating the peak at 723 eV. By including both Fe_3_O_4_ and Fe^2+^, a more accurate L_2_-edge fit was created, whilst maintaining the fit at the L_3_-edge. However, calculating a precise fit and percentage value for the phases contributing to the F2 spectrum was difficult, as the spectrum displayed signs of saturation, manifesting as an enhanced post-edge absorption intensity when compared to the fitted data.

The spectrum from F3 showed subtle changes in L_3_-edge features, with the low energy 708 eV peak becoming less discernible (in comparison to the ferric standard) appearing as a shoulder on the 709.5 eV peak feature. A shoulder feature on the principal Fe^3+^ cation feature is characteristic of the mixed-valence mineral magnetite (a magnetite reference spectrum can be seen in Fig. 2h). The F3 spectrum fit showed this iron inclusion to be comprised primarily of magnetite (*ca.* 60%) with a minor contribution from Fe^3+^ cations. The iron L_2,3_ absorption edge spectrum from the small dense iron deposit labelled F4 in Fig. 5d is shown as the uppermost spectrum in Fig. 5f. This deposit was shown to be in a chemically reduced state as the low energy peak feature at 708 eV was not discernible, appearing as a shoulder on the 709.5 eV peak feature, again consistent with the redox-active mixed-valence mineral magnetite. The fit of the F4 spectrum showed this area to be principally comprised of magnetite (*ca.* 81%), with minor contributions from Fe^3+^ and Fe^2+^ (see ESI Fig. S6† for the calculated iron components used for fitting). The observation of magnetite in APC is consistent with observations by other techniques.^36^,^56^,^57^

Evidence of chemically reduced, redox-active iron phases was also observed in case X from the APC displayed in Fig. 3 (see also Fig. 5g). From the iron L_2,3_-edge X-ray absorption spectra shown in Fig. 5h, the two iron deposits observed in this plaque core were heavily reduced, resembling a ferrous mineral phase, whilst also displaying features consistent with zero valence (Fe^0^) iron.^58^,^59^ Whilst the principal iron L_3_-edge absorption feature for both Fe^2+^ and Fe^0^ resides at 708 eV, these two phases are easily distinguishable by the broader line-shape of the spectrum for Fe^0^ which lacks the multiplet fine structure seen in the oxide spectra, as well as the more prominent L_2_ peak and post-L_2_ edge absorption intensity for Fe^0^ (see for example Fig. S7†).

The fit for region H1 determined this area to be *ca.* 60% comprised of a spectrum resembling Fe^0^ with further contributions from Fe^2+^ and Fe^3+^ cations (see ESI Fig. S7† for the calculated components used for fitting). The evidence for the presence of Fe^0^ is particularly convincing at the iron L_2_-absorption edge where this area strongly resembles the Fe^0^ reference. Likewise, fitting of region H2 indicated a spectrum resembling Fe^0^ to be the predominant phase. The presence of iron in such low oxidation states was unexpected; this is the first time evidence consistent with Fe^0^ has been reported in human APC to our knowledge, confirming the sensitivity of X-ray spectromicroscopy as a tool to probe chemical species within complex material of biological origin.

**Fig. 6:**
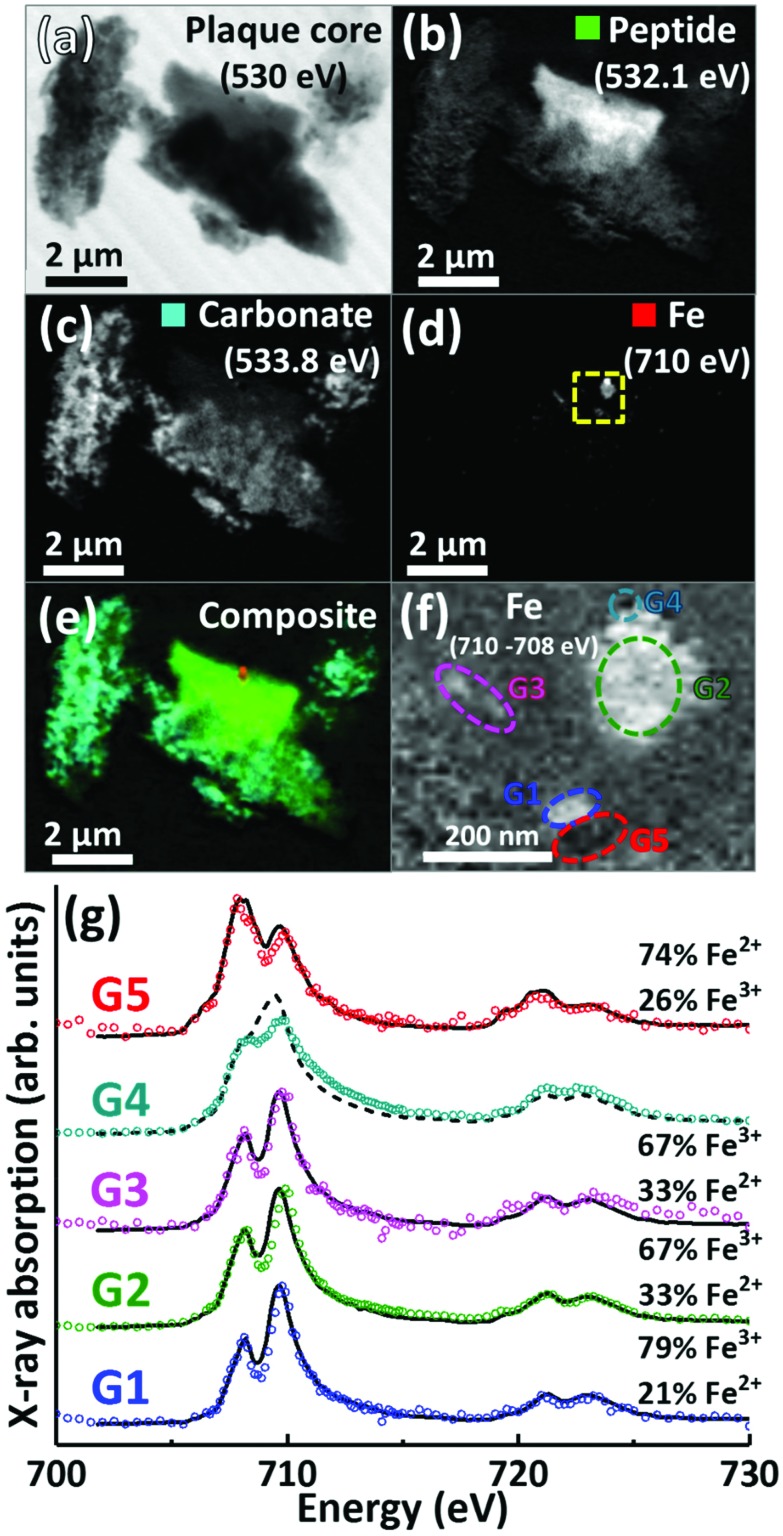
STXM images, speciation dependent contrast maps and iron L_2,3_-edge absorption spectra from an amyloid plaque core from case Y. (a) Off resonance 530 eV image. (b) Oxygen K-edge protein map. (c) Oxygen K-edge carbonate map. (d) Iron L-edge map. (e) Composite image showing: protein (green), carbonate (sky blue) and iron (red) content. Scale bars = 2 μm. (f) High magnification iron oxidation state difference map of the inset area (yellow dashed line) in (d) showing Fe^3+^ (white), and Fe^2+^ (black) content of the iron deposits. Scale bar = 200 nm. (g) Iron L_2,3_-edge absorption spectra from the iron regions labelled in the iron oxidation state difference map (f). The solid and dashed black lines for the spectra correspond to best fit curves created by superposition of suitably scaled iron reference X-ray absorption spectra, see Fig. S1.†

A further APC from case Y is presented in Fig. 6. Speciation dependent mapping showed this plaque similarly comprised of peptides (Fig. 6b) and carbonate (Fig. 6c), with iron content primarily confined to a sub-micron region in the centre of the plaque (Fig. 6d). A high magnification iron oxidation state difference map was created for this region to specifically differentiate iron species by subtracting the image acquired at the prominent Fe^2+^ peak (708 eV) from the image taken at the prominent Fe^3+^ peak (710 eV), resulting in Fe^3+^ deposits showing a bright contrast and Fe^2+^ a dark contrast. This oxidation state difference map revealed localised variation in Fe^3+^ and Fe^2+^ content even within single nanoscale iron deposits (Fig. 6f). Examination of specific X-ray absorption across the iron L_2,3_-edge (Fig. 6g) revealed significant variation in the oxidation state across this area containing particulate iron (Fig. 6f), with evidence of primarily ferric iron (areas G1, G2 and G3) and a Fe^2+^ dominated spectrum (area G5) consistent with a largely ferrous material (*ca.* 74%) as determined by the fitting of the spectra. Area G4 shows evidence of saturation effects at the L_3_-edge, resulting in an artificially high shoulder feature on the peak feature at 709.5 eV, giving the false impression of a high Fe^2+^ content. An attempted fit for this spectrum is provided as a dashed black line in Fig. 6g; however, due to the saturation effects, a quantitative analysis of the constituent iron phases in this area was not possible.

**Fig. 7:**
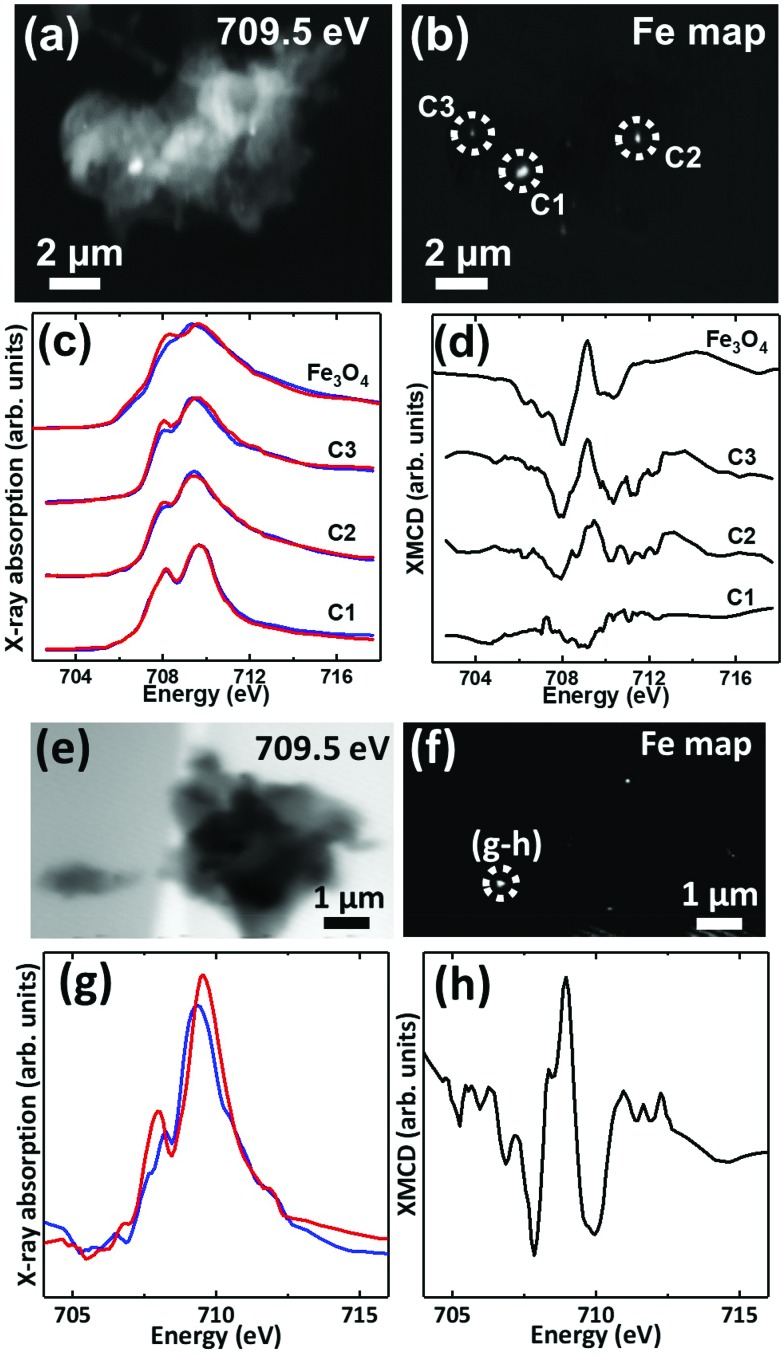
(a, e) X-ray microscopy images (b, f) iron L_3_ edge speciation maps (c, g) X-ray absorption spectra and (d, h) XMCD spectra from an APC of case X. Panels (c) and (g) show X-ray absorption spectra obtained using LCP (blue spectra) and RCP (red spectra). Panels (d) and (h) show the corresponding XMCD spectra created by subtracting the RCP spectra from the LCP spectra. All spectra were obtained in a magnetic field of ∼150 mT applied parallel to the incident X-ray beam.

Iron L_3_-edge X-ray microscopy images, speciation maps, circular polarization-dependent X-ray absorption and XMCD spectra from case X APC sections (adjacent to those shown in Fig. 3 and 5(g, h)), measured under an applied magnetic field are displayed in Fig. 7. Iron L_3_-edge X-ray absorption and XMCD spectra from a reference magnetite standard (labelled Fe_3_O_4_) examined on the same beamline are shown in Fig. 7c and d. The magnetite XMCD spectrum displays a characteristic 3-point negative–positive–negative peak structure corresponding to magnetic iron cations present in Fe^2+^ octahedral, Fe^3+^ tetrahedral and Fe^3+^ octahedral sites respectively (see Telling *et al*., 2017^46^). Thus the appearance of these features in XMCD spectra obtained from APC would indicate the presence of ordered magnetite. Conversely, the absence of XMCD peak features (*i.e.* where the spectra obtained under RCP and LCP are identical) would indicate the presence of a non-magnetic iron phase.

XMCD spectra obtained from three iron-rich inclusions highlighted in Fig. 7b are displayed in Fig. 7d. By examining these spectra, a clear difference in magnetic ordering can be observed across the areas. Area C1 shows a weak XMCD effect, with a single poorly defined negative peak, whereas areas C2 and C3 display XMCD features consistent with magnetite. In particular, the XMCD spectra from area C3 displays a 3-point negative–positive–negative peak structure consistent with the magnetite reference in terms of both shape and relative peak intensities. Likewise, the examination of an iron inclusion from a further case X APC shown in Fig. 7(e–h), provided an XMCD spectrum consistent with a magnetite-like phase. However, in this case the relative positive and negative peak intensities in the XMCD spectrum (Fig. 7h), as well as the shape of the X-ray absorption spectra (Fig. 7g) suggest a more oxidised form similar to maghemite (see Telling *et al*.^46^). Taken together these data strongly indicate the presence of varying oxidation states of the mineral magnetite within APC from human AD tissue.

**Fig. 8:**
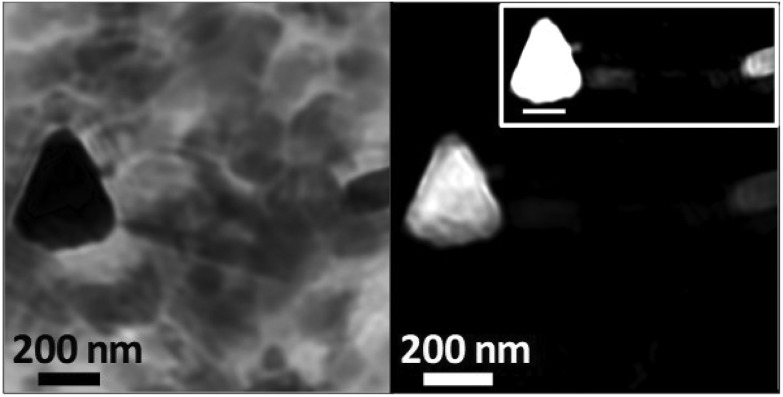
Ptychography image (710 eV; left) and iron contrast map (right) of iron deposit 2 located in the amyloid plaque core of Fig. 5(a–e). High contrast iron map (right; inset) shows additional iron detail in this region.

To examine the morphology of iron deposits in APC in even greater detail, high-resolution ptychographic imaging was employed. Ptychography involves scanning a sample and simultaneously collecting scattered X-rays in addition to transmitted X-rays, thereby allowing a much greater spatial resolution to be resolved (*ca.* 2 nm) compared to traditional STXM techniques (20–30 nm in spatial resolution).^60^Fig. 8 shows a ptychography image and iron content map from iron region 2 of the APC shown in Fig. 5(a–e). A dense triangular shaped object *ca.* 300 nm in diameter can be seen in the left field of the 710 eV image. Iron mapping showed the entirety of the triangular deposit to contain iron, with the structure strongly resembling the morphology of magnetite biominerals previously observed in magnetotactic microorganisms^61^ and extracted from human brain tissue.^62^,^63^ A single crystal of magnetite this large would be magnetically blocked. Altering the intensity threshold in the iron contrast image (see Fig. 8, inset) revealed a lower-concentration background of iron distributed beyond the triangular deposit, indicating the presence of iron throughout the APC in addition to the dense iron deposits. Elongated (rod-like) structures are also observed in the 710 eV image (Fig. 8a), but not in the iron map, that are consistent with the size/morphology of nanocrystalline calcium-based minerals such as calcite^64^ or hydroxyapatite,^65^ supporting the earlier interpretation of the calcium and carbonate maps seen in all the APC.

## Discussion

The precise analysis achieved here with X-ray spectromicroscopy and X-ray magnetic circular dichroism revealed APC associated with diffuse iron, and dense iron deposits incorporating ferrous iron, as well as the mixed-valence iron oxide magnetite. In addition, evidence consistent with zero-valent iron was observed in these structures for the first time. Calcium deposits were also observed within APC, including novel evidence of plaque calcification and calcium carbonate deposition. The presence of these iron and calcium features was observed consistently in multiple plaques isolated from the two independent AD cases.

The incorporation of iron into APC is in agreement with previous examination of human AD tissues, for example by histology,^21^ micro particle-induced X-ray emission analysis,^34^ and MRI.^35^ The present spectromicroscopy observations in human APC are supported by our recent *in vitro* Aβ/iron^24^,^25^ and *ex vivo* transgenic APP/PS1 mouse X-ray spectromicroscopy studies.^46^,^66^ In the APC presented here, iron was principally evident as sub-micron dense deposits, with no direct correlation between peptide and iron morphology being observed. These findings are consistent with observations of iron particulates in regions of dense amyloid pathology with an apparently amorphous structure.^46^ For both AD cases investigated, iron L_2,3_-edge X-ray absorption spectra from APC demonstrated iron to be present in multiple different oxidation states. These ranged from pure ferric phases (Fe^3+^), to the mixed-valence (Fe^2+^/Fe^3+^) magnetic phase magnetite, predominantly ferrous (Fe^2+^) and, outstandingly, spectra consistent with zero-valent (Fe^0^) materials. Importantly, this variation in Fe oxidation state occurred across individual APC, and within single nanoscale deposits, a result in keeping with our STXM examination of transgenic mouse APP/PS1 tissue.^46^

Our recent *in vitro* studies used X-ray spectromicroscopy to demonstrate that aggregation of synthetic Aβ(1–42) is accompanied by chemical reduction of ferric iron into a pure ferrous form.^24^,^25^ Earlier studies indicated redox cycling of iron in the presence of Aβ,^26^,^28^ further supported by evidence for mixed valence iron oxide in an APP/PS1 mouse model of AD.^66^ These new findings in human APC support the hypothesis of a dynamic processes occurring *in vitro* and *in vivo*, and strongly implicate Aβ in the formation of elevated levels of potentially redox-active ferrous and zero-valent iron phases in human brain. Interactions of Aβ with Fe ions can occur by coordination through His residues in the N-terminal region, as observed by Raman scattering.^67^ In prior work it was suggested that Aβ becomes oxidized in the process of reducing iron by residue Met-35 of Aβ(1–42).^68^,^69^ Recently, however, it has been shown that Met-35 is inaccessible once amyloid fibres have formed, since this residue is buried in a hydrophobic interface region.^70^ The X-ray spectromicroscopy measurements performed here permitted unambiguous identification of the redox state of a variety of iron species, but did not provide information about the oxidation state of the Aβ in the APC. In the context of our prior *in vitro* analysis, this evidence for a ferrous and zero-valent iron fraction also occurring in the APC is noteworthy because it indicates the stability of the analyte during sample handling. These APC were necessarily resin-embedded and sectioned prior to analysis, which was not required for synchrotron X-ray analysis of the aggregates formed *in vitro* that also evidenced chemical reduction of iron in the presence of aggregating Aβ.^24^,^25^

The ability of Aβ to cycle iron throughout the ferrihydrite–magnetite–wüstite and potentially even zero-valent iron phase paradigm *in vivo*, implicates aggregating Aβ in the *sustained* generation of free-radical-producing iron species. The catalytic behaviour of iron in Fenton chemistry and related processes means that the free radical burden is likely to be more influenced by local iron chemistry than by absolute iron concentration. In the AD brain with evidence for disrupted iron metabolism and localised iron accumulation, there would be no shortage of fuel for these reactions; the localised nature of redox-active iron formation would have the potential to catalyse generation of free radical burdens inducing neuronal damage/death over time. Indeed, increased levels of oxidative stress have previously been reported in tissues with a high density of Aβ deposition,^69^ although the capacity of amyloid to directly generate free radicals has often been debated.^71^,^72^ From our observations we suggest that Aβ is acting *indirectly* rather than *directly* in this regard, with free radicals generated through Aβ conversion of redox-inactive iron phases into redox-active forms.

Gaining a better understanding of the impacts on iron biochemistry of aggregating Aβ *versus* established APC remains a pressing issue, emphasized in recent work showing that Aβ immunization increased iron deposition in the choroid plexus.^73^ The debate continues as to whether APC formation has a protective effect through lowering the availability of unbound redox-active metal ions, and whether this protective effect may offset free radical damage arising indirectly from Aβ fibril formation. One explanation is that the aggregating mono- or oligomeric Aβ is a driver of free radical generation through its chemical reduction of iron,^24^ and that subsequent formation of dense insoluble aggregates may serve a protective role in having sequestered (effectively chelated) the redox-active iron species typical of those reported here in the APC. This is consistent with the hypothesis that Aβ plaques may be a physiological response rather than a pathological process in their own right.^74^ However, the nanoscale X-ray spectromicroscopy analysis presented here indicates that iron is present in a range of oxidation states, which could indicate the dynamic redox-cycling occurring within APC upon metal overload; in this scenario, dissolution of plaques may create local sources of toxic reactive iron species in the brain.

Ptychography obtained at the iron L_3_-edge enabled the morphology of iron deposits within APC to be resolved at a remarkably high spatial resolution of *ca.* 2 nm. Through this approach we identified an iron deposit with a strong resemblance to a single magnetite/maghemite crystal. Further magnetic characterization of iron inclusions within APC using XMCD confirmed the presence of magnetite in multiple plaque cores. This supports prior work suggesting a role for Aβ in the biosynthesis of magnetite in human brain,^36^ where evidence for magnetite has previously been reported in inorganic materials extracted from brain tissue homogenates,^62^,^63^ in isolated ferritin,^38^ in ferritin-core-sized iron oxide deposits located within APC by electron beam methods,^36^ and in plaque-rich human tissue from AD cases.^57^

The precise source(s) of the iron integrated into APC *in vivo* is not yet known. Multiple sources of iron may be relevant to amyloid–iron interaction in AD such as: ferritin-bound ferrihydrite, transferrin, labile iron pools (including jettisoned ferritin iron content^75^), hemosiderin formed at sites of microbleeds and haemorrhage in the brain, from disrupted neuronal mitochondria, and/or potential external sources of iron such as airborne particulate matter which have been suggested to enter the brain *via* the olfactory bulb.^76^ Furthermore, the influence of the initial iron phase upon amyloid's reductant properties *in vivo* has not previously been characterised. Closer examination of the location and characteristics of amyloid/iron structures within intact AD tissues may provide clearer indications as to the source of amyloid–associated iron.

In all APC examined here, extensive accumulation of calcium was observed. By characterizing both calcium carbonate and total calcium content we demonstrated that calcium within APC was present in more than one form. Although the non-carbonate calcium phases observed could not be fully characterised, oxygen K-edge X-ray absorption features indicated this material was comprised of a hydrous calcium phase. Apatite, a calcium phosphate mineral (Ca_5_(PO_4_)_3_), is produced in biological systems and readily associates with water to form hydroxyapatite (Ca_5_(PO_4_)_3_(OH)). It is therefore possible that hydroxyapatite is the crystalline form adopted by the non-carbonate calcium phase observed. Subsequent analysis of the phosphor content of APC would be required to confirm this. Ptychographic images of APC revealed the presence of rod-like features with size and morphology matching calcium-based crystalline phases such as calcite or hydroxyapatite,^64^,^65^ which indicated that calcium biomineralization may be occurring during the formation of amyloid plaques in AD. Importantly, the confirmed presence of multiple calcium phases suggests that a dynamic process of plaque calcification may occur *in vivo*.

From the present experiments it was not possible to determine the origin of the calcium, or the effect of calcium on amyloid/iron interactions (such as competitive calcium/iron binding to amyloid). Examples of potential sources for the calcium observed in APC include: transferrin, in which carbonates are used for iron binding; calmodulin or other calcium binding proteins such as lithostathine (an inflammatory protein shown to accumulate in APC); or pools of extracellular Ca^2+^ used in processes such as neurotransmitter release.^11^,^14^ It is not yet determined if the forms of calcium observed in the APC are representative of the original source(s) of calcium, or if a biomineralization pathway (to be determined) produces carbonate in conjunction with APC formation.

The accumulation of calcium within APC may drive, or arise from, disrupted calcium trafficking and homeostasis in AD patients. Calcium triggers numerous signalling pathways in both excitable and non-excitable brain cells, whilst also regulating synaptic connections.^11^,^14^ In prior work, Aβ impacted calcium signalling pathways to the detriment of neuronal health and function.^1^ Maintaining equilibrium in the extracellular Ca pool is vital to sustain these calcium signalling pathways, so the binding of calcium by Aβ as evidenced in this present study may have a detrimental effect upon Ca-dependent cellular signalling arising from the propensity of Aβ to act as a metal-binding protein.

Concurrent deposition of iron and calcium has previously been observed at significantly lower spatial resolution in tissue exhibiting amyloid aggregates (in the thalamus of APP mice^43^,^77^), and in APC from the hippocampus.^43^,^44^ The results presented here are unique in obtaining the iron and calcium distribution, and the chemical speciation and mineral phase of iron and calcium inclusions with nanoscale resolution in human APC from confirmed AD cases. In particular, the appearance of dense calcium carbonate regions, co-located with another calcium phase (potentially based on apatite), was an unprecedented result. One interpretation of the distribution variation of the two calcium phases observed in these APC is that the transformation from apatite-like phases to calcium carbonate may occur over time. Detailed investigation of such processes, to predict age-dependent characteristics of calcifications *in vivo*, will help determine if features such as calcifications have utility as markers of disease progression to aid with clinical staging. Notably, “ferro-calcic” amyloid plaques have been evidenced in magnetic resonance imaging (MRI) contrast in the thalamus of transgenic APP/PS1 mice,^77^ where the MRI properties of dense iron and calcium-rich deposits are sufficiently different from the surrounding tissue that they provide endogenous contrast. It will be important to distinguish calcium from iron deposition if the impact of iron modifying treatments is to be evaluated clinically by MRI.

The roles of iron and calcium cannot be fully explored without consideration of the wider range of metal and metalloid elements detected in human APC, including other transition metals (*e.g.* copper, zinc, manganese), aluminium (non-essential, and which facilitates iron-mediated oxidative reactions as well as affecting Aβ aggregation), and silicon, amongst others.^34^,^36^,^44^,^78^ In semi-quantitative synchrotron XRF analysis of the transition metal burden in human APC, copper concentration was elevated to the greatest extent (relative to copper concentration in the surrounding tissue) and linked with elevated production of H_2_O_2_, a key component of Fenton chemistry. The proportionate increase in iron and calcium was comparatively modest for these more abundant elements.^44^ To provide effective neuroprotection against toxicity arising from amyloid–metal reactions, a better understanding of the many competing interactions and influence of co-factors on reaction rates, including the chemically bound and unbound forms of each species, is required. For example, labile iron is more chemically available to partake in redox chemistry than complexed iron species (*e.g.* Fe^2+^ and Fe^3+^ of haemoglobin), and would be readily reduced in the presence of oxidants such as the H_2_O_2_ associated with copper loading in AD plaques, fuelling the catalytic production of ROS by iron.^44^,^79^

With iron being essential to healthy brain function, especially for energy production in mitochondria, it is of paramount importance to determine how to distinguish normally-metabolized iron from any iron species that elevates neuronal stress. To date, therapies that target iron metabolism in AD have been unsuccessful. One reason may be the lack of specificity resulting in depletion of iron stores and other essential metal cations required to sustain neuronal health (Cu, Zn, Mg, among others).^8^,^80^ This study represents a significant advance in the spatial resolution and precise speciation with which various iron phases are described in APC from AD cases. As different iron phases have distinct physicochemical properties, these findings may prove vital in the tailoring of AD diagnostics and therapies that discriminate detrimental forms of iron from those that are essential to normal function.

## Conclusions

The X-ray spectromicroscopy methodology developed in this study enabled characterization of the distribution of organic materials (proteins), and precise nanoscale imaging and speciation of inorganic materials (iron and calcium compounds) in APC. The STXM methods enabled this to be done without the need for chemical fixation or contrast agents that significantly affect metal chemistry, and with significantly lower beam dose than required for equivalent electron-beam analyses. The unique concurrent characterization of iron and calcium within human amyloid plaques is a finding that will enable progress in understanding the implications of interactions between amyloid-β, calcium, and iron, where disrupted calcium signalling pathways and elevated intracellular calcium have previously been observed in Alzheimer's disease. These iron and calcium species observed within the APC are assumed to be the products of metal–amyloid interactions *in vivo*, where wider evidence points to these interactions playing a role in the progression of AD.

The results support the hypothesis that iron is chemically reduced in the presence of aggregating Aβ and implicate this process as source of excess free radical generation. Whereas it has previously been assumed that these chemically reduced iron phases are rich in ferrous iron (Fe^2+^), we have now found evidence consistent with the presence of both zero-valent (Fe^0^) as well as ferrous-rich iron phases within pathological Aβ structures. These observations do not completely exclude the possibility of other phases such as iron sulphide.^81^ In addition, through detailed magnetic characterization, we demonstrate the mixed valence magnetic iron phase magnetite to be present within APC. Furthermore, we provide direct evidence that APC have the capacity to bind large quantities of calcium-rich species; this may be a significant sign of disrupted calcium homeostasis and cellular signalling, resulting in neuronal deterioration over time. The new observation that multiple calcium phases are present in APC suggests that a dynamic process of plaque calcification may occur *in vivo*.

Importantly, this unique application of X-ray spectromicroscopy has enabled concurrent *in situ* nanoscale characterization of iron and calcium minerals in human APC. These new observations support the hypothesis that Aβ plays a major role in disrupted iron and calcium biochemistry, and raise questions about whether Aβ binding enhances or counteracts the increased oxidative burden and disrupted neuronal signalling evidenced in AD. Determining the key mechanisms governing the formation of APC and neuronal responses to these metal–amyloid phases has scope to facilitate improved diagnosis of AD, as iron and calcium minerals affect magnetic resonance imaging signals. It also offers new perspectives for the development of therapies that successfully target iron toxicity in Alzheimer's disease patients.

## Conflicts of interest

There are no conflicts to declare.

## Supplementary Material

Supplementary information
