# Supplementary material for: Nanoscale synchrotron X-ray speciation of iron and calcium compounds in amyloid plaque cores from Alzheimer's disease subjects
Source: Nanoscale. 2018 Apr 24;10(25):11782–96. doi: 10.1039/c7nr06794a (PMC6034173; doi:10.1039/c7nr06794a)

Electronic Supplementary Material (ESI) for Nanoscale.
This journal is © The Royal Society of Chemistry 2018

Supplementary figures

Figure S1. Scaled iron $L_{2,3}$-edge absorption reference spectra used to fit the amyloid plaque core iron spectra described in the main text.

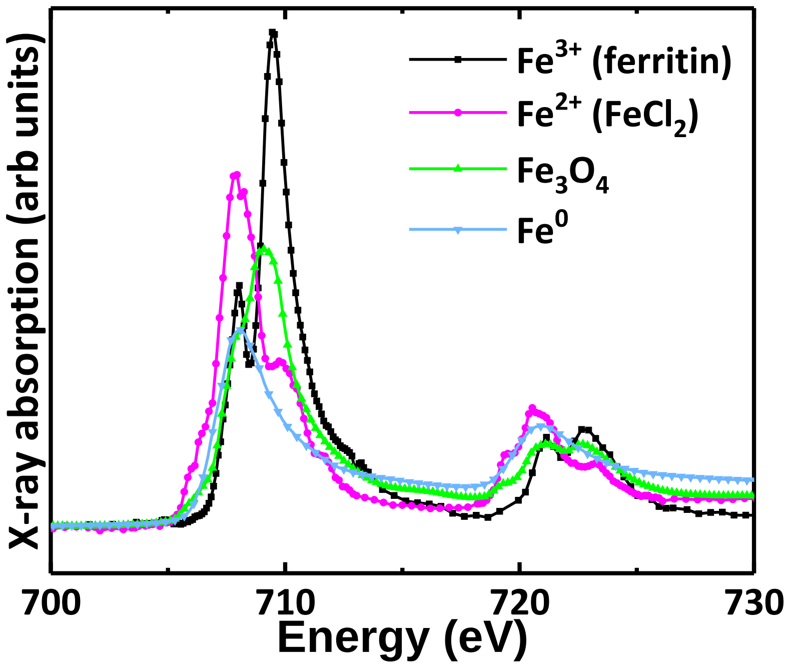

Figure S2. Positive correlation between x-ray microscopy peptide maps taken at the carbon *K*-absorption edge (left) and oxygen *K*-absorption edge (right) absorption edges of a plaque core taken from case Y. Section thickness = 500 nm.

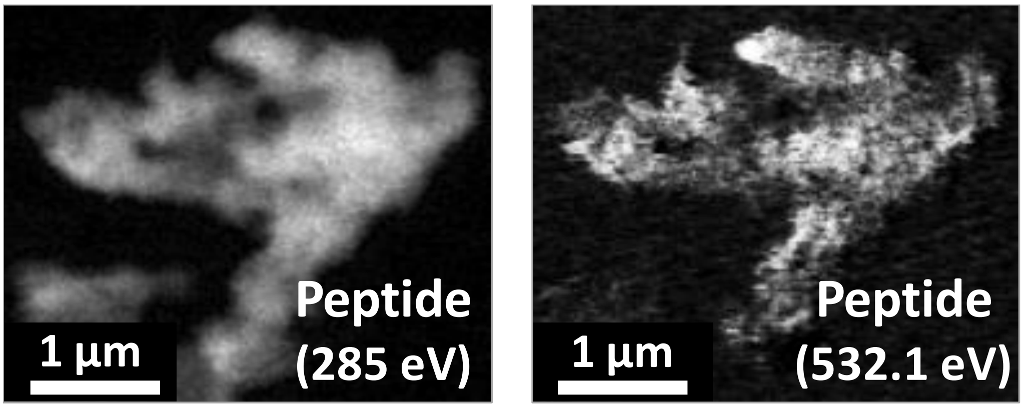

Figure S3. X-ray microscopy images taken at 530 eV, displaying the variation in amyloid plaque core size. From AD case Y. Section thickness = 500 nm.

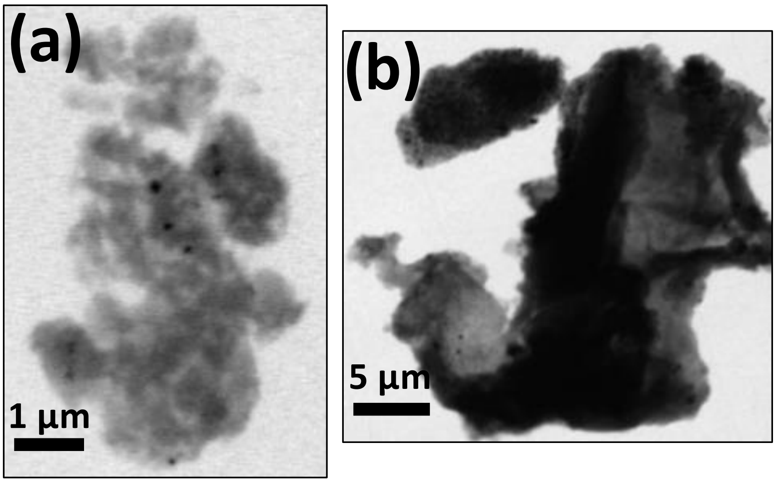

Figure S4. Comparisons of x-ray microscopy peptide maps obtained from amyloid plaque cores 200 nm (left) and 500 nm (right) in thickness. 200 nm and 500 nm sections are from cases X and Y respectively.

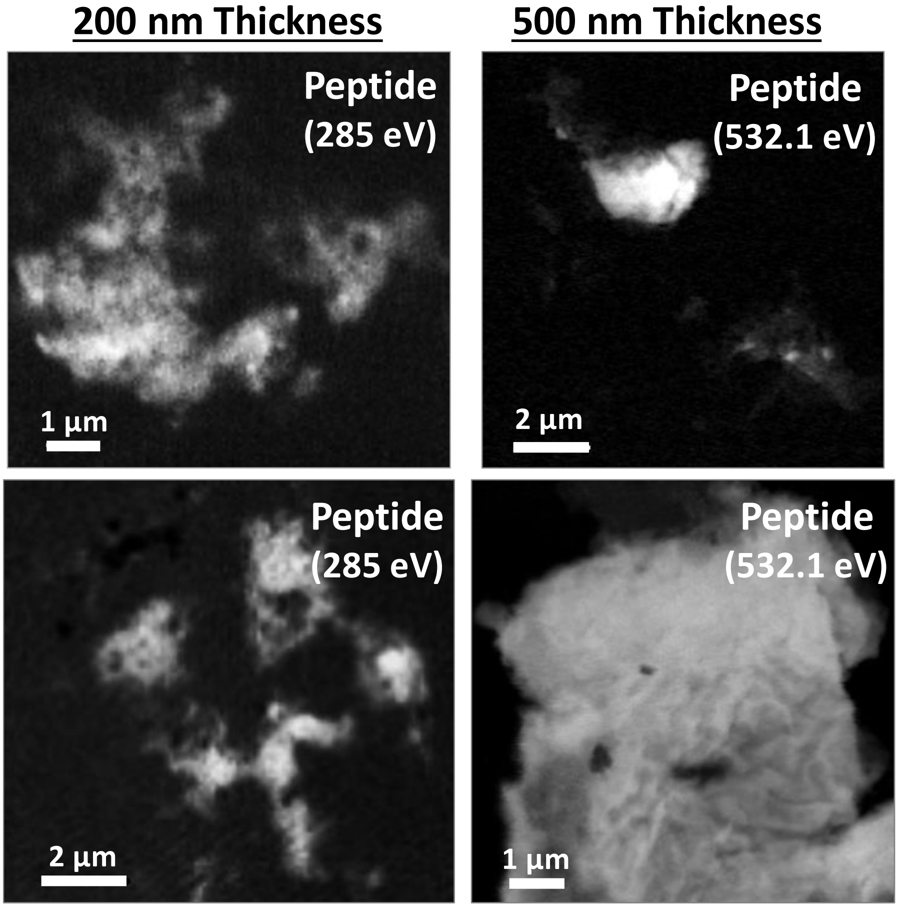

Figure S5. X-ray microscopy images and speciation dependent maps of amyloid plaque cores from case Y.

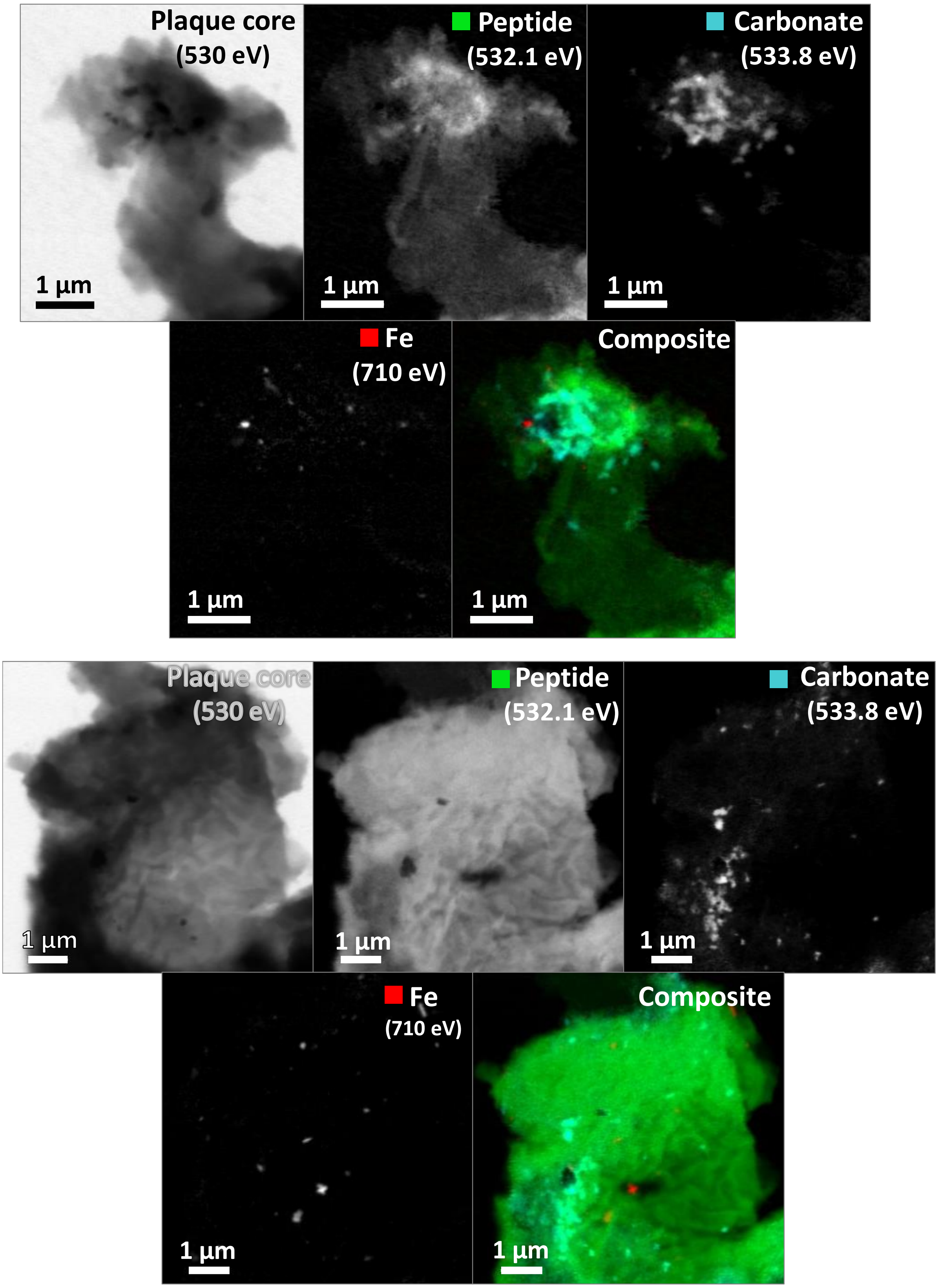

Figure S6. Relative intensities of the iron $L_{2,3}$-edge absorption reference spectra used to create a fit for spectrum F4 from Figure 5 in the main text.

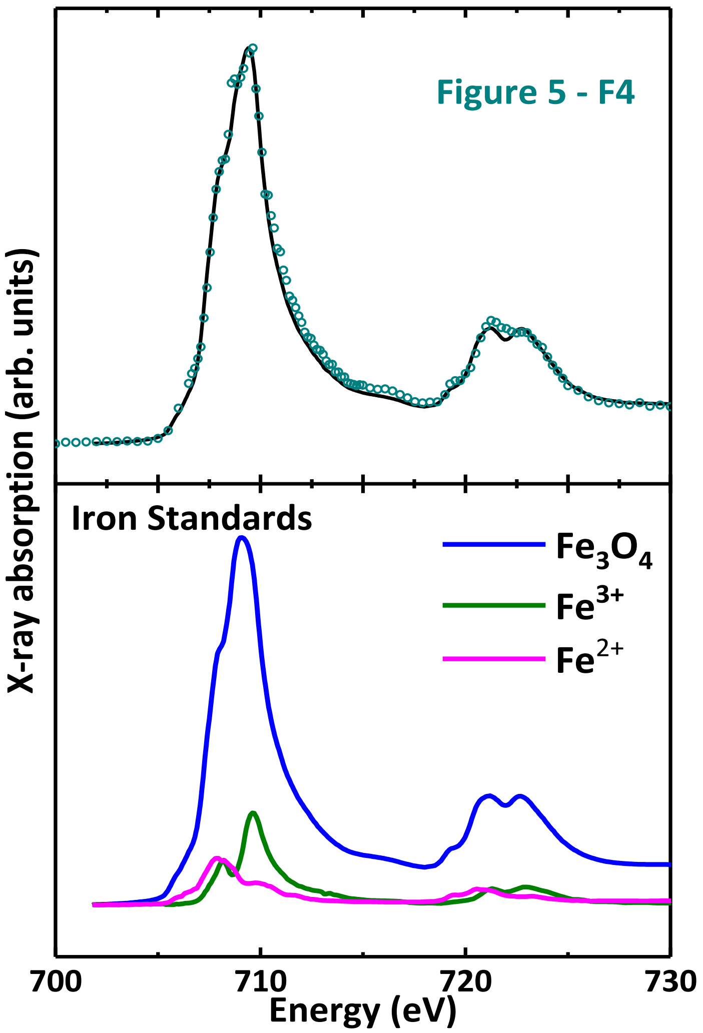

Figure S7. Relative intensities of the iron $L_{2,3}$-edge absorption reference spectra used to create a fit for spectrum H1 from Figure 5 in the main text.

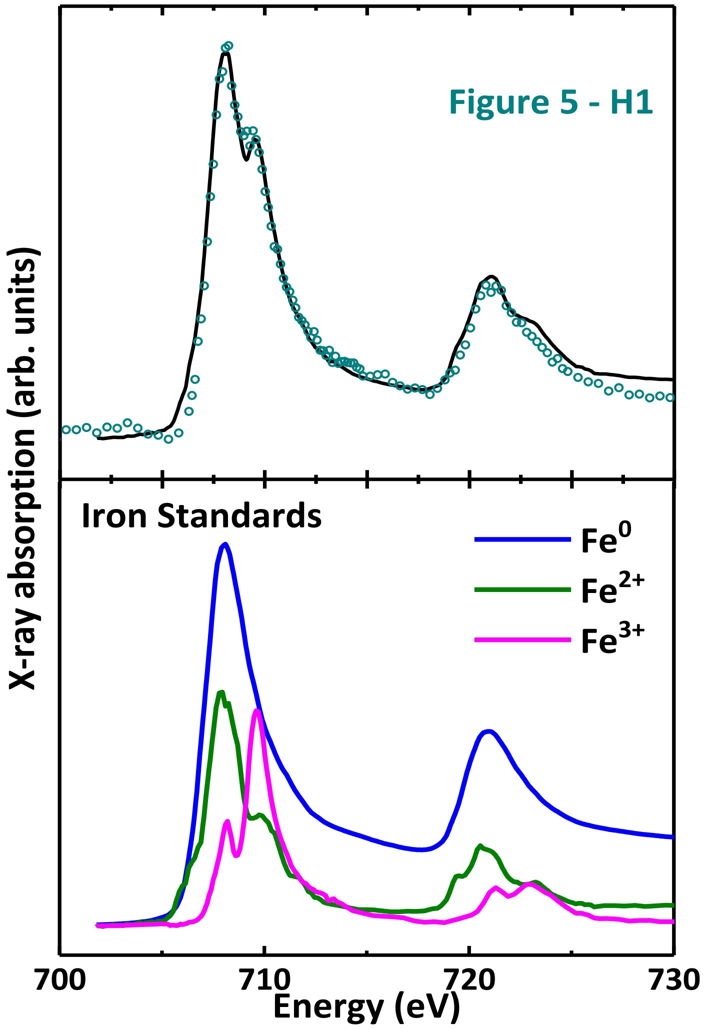

Supplement: Supplementary file 1 [file NR-010-C7NR06794A-s001.pdf]
